# Heterozygous eNOS deficiency is associated with oxidative stress and endothelial dysfunction in diet‐induced obesity

**DOI:** 10.14814/phy2.12630

**Published:** 2015-12-10

**Authors:** M. Irfan Ali, Xunsheng Chen, Sean P. Didion

**Affiliations:** ^1^ Vascular Biology CenterGeorgia Regents UniversityAugustaGeorgia; ^2^ Department of Pharmacology and Department of NeurologyThe University of Mississippi Medical CenterJacksonMississippi

**Keywords:** Carotid artery disease, diabetes mellitus, type 2, genetically altered mice, haploinsufficiency, high fat diet, Interleukin‐6, *NOS3*

## Abstract

Heterozygous endothelial nitric oxide synthase (eNOS) deficiency is associated with normal endothelium‐dependent responses, however, little is known regarding the mechanisms that maintain or impair endothelial function with heterozygous eNOS deficiency. The goals of this study were to (1) determine mechanism(s) which serve to maintain normal endothelial function in the absence of a single eNOS gene; and (2) to determine whether heterozygous eNOS deficiency predisposes blood vessels to endothelial dysfunction in response to a high‐fat diet (HFD). Responses of carotid arteries were examined in wild‐type (*eNOS* ^*+/+*^) and heterozygous eNOS‐deficient (*eNOS* ^*+/−*^) treated with either vehicle (saline), N^G^‐nitro‐L‐arginine (L‐NNA, 100 μmol/L), an inhibitor of nitric oxide synthase, or 1H‐[1,2,4]oxadiazolo[4,3‐a]quinoxalin‐1‐one (ODQ, 1 μmol/L), an inhibitor of soluble guanylyl cyclase (sGC), and in *eNOS* ^*+/+*^ and *eNOS* ^*+/−*^ mice fed a control (10%) or a 45% HFD (kcal from fat). Responses to acetylcholine (ACh) were similar in vehicle‐treated arteries from *eNOS* ^*+/+*^ and *eNOS* ^*+/−*^ mice, and were equally inhibited by L‐NNA and ODQ. Phosphorylation of eNOS Ser1176, a site associated with increased eNOS activity, was significantly greater in *eNOS* ^*+/−*^ mice most likely as a compensatory response for the loss of a single eNOS gene. In contrast, responses to ACh were markedly impaired in carotid arteries from *eNOS* ^*+/−*^, but not *eNOS* ^*+/+*^, mice fed a HFD. Vascular superoxide levels as well as plasma levels of the pro‐inflammatory cytokine interleukin‐6 (IL‐6) were selectively increased in HFD‐fed *eNOS* ^*+/−*^ mice. In reconstitution experiments, IL‐6 produced concentration‐dependent impairment of endothelial responses as well as greater increases in NADPH‐stimulated superoxide levels in arteries from *eNOS* ^*+/−*^ mice fed a control diet compared to *eNOS* ^*+/+*^ mice. Our findings of increased Ser1176‐phosphorylation reveal a mechanism by which NOS‐ and sGC‐dependent endothelial function can be maintained with heterozygous eNOS deficiency. In addition, heterozygous eNOS deficiency predisposes blood vessels to developing endothelial dysfunction in response to a HFD. The impairment produced by a HFD in *eNOS* ^*+/−*^ mice appears to be mediated by IL‐6‐induced increases in vascular superoxide. These findings serve as an important example of eNOS haploinsufficiency, one that may contribute to the development of carotid artery disease in obese humans.

## Introduction

Nitric oxide derived from endothelial nitric oxide synthase (eNOS) represents an important homeostatic mechanism that maintains a number of functions within blood vessels, including endothelium‐dependent relaxation (Beckman and Koppenol 1996; Thomas et al. 2003; Pacher et al. 2007). Bioavailability of nitric oxide is influenced by several factors, but most notably nitric oxide levels are most reflective of eNOS expression and activity as well as by expression and activity of superoxide‐generating enzymes such as NADPH oxidase (Konior et al. 2014). Functionally, increases in vascular superoxide can have profound effects on vascular function as evidenced by reductions in endothelium‐dependent relaxation produced by stimuli known to increase superoxide, such as angiotensin II, ceramide, and NADPH (Didion and Faraci 2002; Didion and Faraci. 2005, 2007; Schrader et al. 2007). Homozygous eNOS deficiency is associated with the loss of endothelial responses to acetylcholine in a number of different blood vessels (Huang et al. 1995; Shesely et al. 1996; Faraci et al. 1998; Chataigneau et al. 1999; Waldron et al. 1999). In contrast, heterozygous eNOS deficiency is associated with normal endothelial responses (Lamping and Faraci 2001). These findings suggest that the presence of a single eNOS gene is sufficient to maintain normal vascular responsiveness under baseline conditions to endothelium‐dependent agonists such as acetylcholine. Somewhat surprisingly, however, there is very little known regarding the mechanisms that contribute to endothelial function in *eNOS* ^*+/−*^ mice. Thus, the first goal of this study was to determine the mechanism(s) that contribute to the maintenance of endothelial function in the absence of a single *eNOS* gene.

The prevalence of obesity worldwide has increased dramatically over the last several decades (Swinburn et al. 2011). It is estimated that nearly a third of US adults are currently overweight or obese (Flegal et al. 2012). Obesity is associated with a significantly higher all‐cause mortality and an increased risk of vascular disease and cardiovascular events, such as carotid artery disease and stroke (Eckel et al. 2004; Fox et al. 2007; Bodenant et al. 2011; Flegal et al. 2013). In addition, obesity is associated with endothelial dysfunction in a number of animal models of obesity as well as obese humans (Didion et al. 2005, 2007; Dobrian et al. 2001; Keaney et al. 2003). Oxidative stress coupled with reductions in nitric oxide bioavailability appear to contribute to endothelial dysfunction in obesity (Didion et al. 2005, 2007; Dobrian 2001; Keaney et al. 2003; Lynch et al. 2013; Molnar et al. 2005; Phillips et al. 2005). Although a number of studies have shown that increases in superoxide contribute to endothelial dysfunction in obesity, there is little to no information related to whether inherent reductions in eNOS expression and/or activity, such as that which may occur with polymorphisms in the promoter region of the eNOS gene (Doshi et al. 2010), predisposes blood vessels to the development of endothelial dysfunction with obesity. Thus, the second goal of this study was to test the hypothesis that heterozygous eNOS deficiency is associated with a greater susceptibility to developing endothelial dysfunction and obesity in response to a high‐fat diet (HFD). As oxidative stress and increases in inflammatory markers are associated with endothelial dysfunction, we also examined the relationship between heterozygous eNOS deficiency and obesity on NADPH‐derived superoxide levels, levels of the inflammatory cytokine interleukin‐6 (IL‐6), and endothelial dysfunction.

## Materials and Methods

### Experimental animals

Male wild‐type (*eNOS* ^*+/+*^; C57BL/6J; #000664) and heterozygous eNOS‐deficient (*eNOS* ^*+/−*^) mice were studied. *eNOS* ^*+/−*^ mice were generated by breeding male *eNOS* ^*−/−*^ mice (B6.129P2‐*Nos3* ^*tm1Unc*^/J; #002684) with female C57BL/6J (*eNOS* ^*+/+*^; #000664) mice (Shesely et al. 1996). *eNOS* ^*−/−*^ mice used in the breeding strategy had been backcrossed at least 10 times to C57Bl/6 mice and thus were congenic. This is important as C57Bl/6 mice have been a strain that has been shown to be particularly sensitive to the development of obesity and type 2 diabetes when placed on a HFD (Rebuffe‐Scrive et al. 1993; Surwit et al. 1995; Brownlow et al. 1996; Collins et al. 2004; Lynch et al. 2013).

Mice were fed either a control diet (10% kcal from fat; #D12450B; Research Diets, New Brunswick, NJ) or a HFD (45% kcal from fat; #D12451) for 30 weeks beginning at 6–8 weeks of age. Systolic blood pressure was measured using tail‐cuff plethysmography (Visitech Systems BP‐2000, Apex, NC) as described previously 1 week prior to conclusion of the experimental diet (Schrader et al. 2007). All experimental protocols conformed to the National Institutes of Health Guide for the Care and Use of Laboratory Animals and were approved by the Institutional Animal Care and Use Committee.

### Vascular function studies in carotid artery

Vascular responses in mouse carotid artery were examined in isolated organ chambers as described previously (Didion et al. 2005; Schrader et al. 2007). Mice were killed with pentobarbital sodium (150 mg/kg ip), and both carotid arteries as well as the aorta (for measurement of vascular superoxide and western blot analysis) were removed and placed in Krebs buffer with the following ionic composition (mmol): 118.3 NaCl, 4.7 KCl, 2.5 CaCl_2_, 1.2 MgSO_4_, 1.2 KH_2_PO_4_, 25.0 NaHCO_3_, and 11.0 dextrose. Loose connective tissue was removed and each carotid artery was cut into two rings each, 3–4 mm in length. Vascular rings were suspended in individual organ baths containing 20 mL Krebs buffer maintained at 37°C and gassed with 95% O_2_/5%CO_2_. The rings were connected to a force transducer to measure isometric tension (contraction and relaxation). Resting tension was increased to reach a final resting tension of 0.25 g, which was determined in preliminary studies to be optimal for these arteries. Following a 45‐min equilibration period, vessels were precontracted (50–60% of maximum) with the thromboxane analog, 9,11‐dideoxy‐11a,9a‐epoxymethanoprostaglandin F2*α* (U46619). After reaching a stable contraction plateau, concentration–response curves were generated to the endothelium‐dependent agonist, acetylcholine (0.01–100 μmol/L) and for the endothelium‐independent agonist, nitroprusside (0.01–100 μmol/L).

In separate studies, we examined the mechanism(s) that mediate relaxation to acetylcholine and nitroprusside in carotid arteries from *eNOS* ^*+/+*^ and *eNOS* ^*+/−*^ mice. To accomplish this goal, vascular responses to acetylcholine and nitroprusside were examined in carotid arteries treated acutely with either vehicle (saline), N^G^‐nitro‐L‐arginine (L‐NNA, 100 μmol/L), an inhibitor of nitric oxide synthase, or 1H‐[1,2,4]oxadiazolo[4,3‐a]quinoxalin‐1‐one (ODQ, 1 μmol/L), an inhibitor of soluble guanylyl cyclase (sGC) (Faraci et al. 1998; Didion et al. 2001a,b). In order to examine the role of superoxide in the impairment of endothelial function produced in response to a HFD, vascular responses were also examined in the presence of vehicle (saline) or Tempol (1 mmol/L). This concentration of Tempol has been shown previously to lower superoxide levels and improve vascular function (Schrader et al. 2007).

### Western blotting

Aorta samples were obtained as described above, and snap frozen in liquid nitrogen and stored at −80°C for examination of protein expression. Western blotting was performed using similar methods as previously described (Lynch et al. 2013). In brief, protein was extracted, loaded equally (30 *μ*g) into precast gels (10% SDS‐PAGE), and then electrophoresed. Following electrophoresis, proteins were transferred to nitrocellulose, blocked (5% milk), and then incubated with primary antibodies for either eNOS (1:250; BD Biosciences, San Jose, CA), P‐1177/79‐eNOS (1:500; P‐1176 in mouse; BD Biosciences), or GAPDH (1:10,000; Novus Biologicals, Littleton, CO). Protein blots were then incubated with the appropriate secondary antibody, incubated with ECL substrate (GE/Amersham, Pittsburg, PA), and visualized by autoradiographic exposure on film and subsequently digitized. All blots were normalized to GAPDH with the exception of Ser1176, which was normalized to total eNOS/GAPDH.

### Measurement of plasma interleukin‐6

Plasma interleukin‐6 levels were measured using a mouse‐specific IL‐6 ELISA as per the manufacturer's instructions (ALPCO Diagnostics, Salem, NH) as described previously (Gomolak and Didion 2014).

#### IL‐6 reconstitution experiments

Carotid arteries and thoracic aortae were incubated in Dulbecco's Modified Eagle Medium (DMEM) with either vehicle (saline) or IL‐6 (100 and 300 nmol/L) for 22 h at 37°C using methods previously described (Schrader et al. 2007). Vascular responses and superoxide levels were then examined in carotid arteries (as described above) and aortae (described below), respectively.

#### Measurement of vascular superoxide

Basal (nonstimulated) superoxide levels were measured in aorta from *eNOS* ^*+/+*^ and *eNOS* ^*+/−*^ mice fed a control or HFD for 30 weeks using lucigenin (5 μmol/L)‐enhanced chemiluminescence as previously described (Didion et al. 2001a,b; Didion and Faraci 2002). In addition, basal (nonstimulated) superoxide levels were measured in aorta from *eNOS* ^*+/+*^ and *eNOS* ^*+/−*^ mice (control diet only) incubated overnight with either vehicle or IL‐6 (100 or 300 nmol/L). NADPH‐stimulated superoxide levels were measured in each vessel by addition of NADPH (1–100 μmol/L). Following measurements of basal or NADPH‐stimulated superoxide levels, we confirmed that the superoxide signal that was measured was in fact due to superoxide by addition of Tempol. Superoxide levels are reported as relative light units (RLU) per second per mm^2^ tissue.

### Pharmacological agents

Acetylcholine, L‐NNA, nitroprusside, NADPH, and Tempol were obtained from Sigma (St. Louis, MO) and all were dissolved in saline. Mouse recombinant IL‐6 was purchased from BD Biosciences. ODQ and U46619 were obtained from Cayman Chemical (Ann Arbor, MI). ODQ was dissolved in DMSO with all subsequent dilutions made with saline. U46619, which was supplied in methyl acetate, was evaporated with 100% nitrogen gas and resuspended in 100% ethanol with subsequent dilutions being made with saline. All other reagents were of standard laboratory grade.

### Statistical analysis

All data are expressed as Mean ± SE. Relaxation to acetylcholine and nitroprusside is expressed as a percent relaxation to U46619‐induced contraction. Comparisons of relaxation and contraction were made using two‐way analysis of variance followed by Bonferroni's multiple comparisons test. All other comparisons were made using either one‐ or two‐tailed unpaired *t*‐tests. Statistical significance was accepted at *P* < 0.05.

## Results

### Responses of carotid arteries from *eNOS* ^*+/+*^ and *eNOS* ^*+/−*^ mice under baseline conditions and following NOS and sGC inhibition

**Figure 1:**
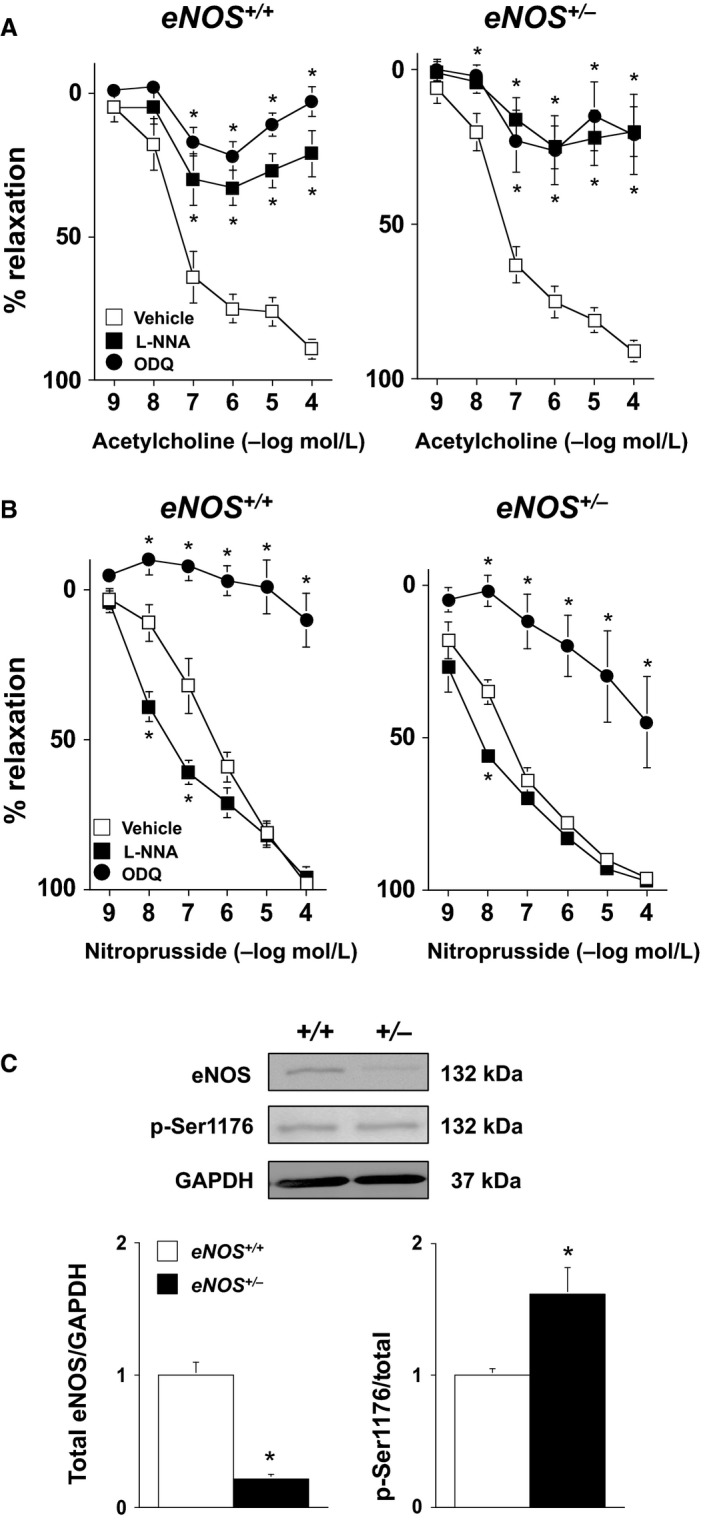
(A and B) Responses to acetylcholine and nitroprusside in carotid arteries from *eNOS* ^*+/+*^ and *eNOS* ^*+/−*^ mice treated with vehicle (saline), L‐NNA (100 μmol/L), or ODQ (1 μmol/L). These findings demonstrate that under baseline conditions heterozygous eNOS deficiency is not associated with alterations in endothelial function despite a 60% reduction in eNOS protein expression. Values are Means ± SE;* n* = 5–8/group; *P < 0.05 versus Vehicle. (C) Western blot and quantification of total eNOS expression and eNOS Ser1176 phosphorylation levels in *eNOS* ^*+/+*^ and *eNOS* ^*+/−*^ mice fed a control diet. As eNOS Ser1176 phosphorylation is an index of eNOS activity, these data provide a mechanism by which endothelial function is maintained with the loss of a single *eNOS* gene. Values are Means ± SE;* n* = 5/group; *P < 0.05 versus *eNOS* ^*+/+*^.

Acetylcholine produced a similar degree (*P* > 0.05) of relaxation in both *eNOS* ^*+/+*^ and *eNOS* ^*+/−*^ mice (control diet fed) under baseline (vehicle) conditions (Fig. 1A). For example, relaxation to 100 μmol/L acetylcholine produced 89 ± 2% and 91 ± 2% relaxation in U46619‐contracted arteries from *eNOS* ^*+/+*^ and *eNOS* ^*+/−*^ mice, respectively. Nitroprusside also produced a similar degree (*P* > 0.05) of relaxation in arteries from both *eNOS* ^*+/+*^ and *eNOS* ^*+/−*^ (i.e., 100 μmol/L nitroprusside produced 98 ± 3% and 96 ± 1% relaxation in arteries from *eNOS* ^*+/+*^ and *eNOS* ^*+/−*^ mice, respectively (Fig. 1B).

As it is not known whether responses of carotid artery in *eNOS* ^*+/−*^ mice are mediated by NOS and sGC like that in *eNOS* ^*+/+*^ mice, we examined the dependency of carotid arteries from *eNOS* ^*+/−*^ mice on NOS‐ and sGC‐dependent mechanisms using a pharmacological approach. In *eNOS* ^*+/+*^ mice, carotid artery responses to acetylcholine were inhibited (*P* < 0.05) by L‐NNA and ODQ (Fig. 1A). For example, acetylcholine (100 μmol/L) produced 89 ± 2%, 21 ± 8%, and 3 ± 5% relaxation in vehicle‐, L‐NNA‐, and ODQ‐treated arteries from *eNOS* ^*+/+*^ mice. In carotid arteries from *eNOS* ^*+/−*^ mice, responses to acetylcholine were also inhibited (*P* < 0.05) by L‐NNA and ODQ (Fig. 1A). For example, acetylcholine (100 μmol/L) produced 91 ± 2%, 20 ± 8%, and 21 ± 13% relaxation in vehicle‐, L‐NNA‐, and ODQ‐treated arteries from *eNOS* ^*+/−*^ mice.

Responses to nitroprusside were enhanced (i.e., leftward shift in the dose‐response curve) in carotid arteries from *eNOS* ^*+/+*^ and *eNOS* ^*+/−*^ mice treated with L‐NNA (Fig. 1B). In *eNOS* ^*+/+*^ mice, ODQ produced marked inhibition of nitroprusside‐induced relaxation. In contrast, there was a greater portion of the nitroprusside response that could not be inhibited with ODQ in *eNOS* ^*+/−*^ mice (Fig. 1B). For example, 100 μmol/L nitroprusside produced 98 ± 3% and 96 ± 1% relaxation in vehicle‐treated, and 10 ± 9% and 45 ± 15% in ODQ‐treated arteries from *eNOS* ^*+/+*^ and *eNOS* ^*+/−*^ mice, respectively.

As NOS appears to mediate acetylcholine‐induced relaxation in both *eNOS* ^*+/+*^ and *eNOS* ^*+/−*^ mice, we next measured the total vascular eNOS protein levels as well as levels of eNOS Ser1176‐phosphorylation, an index of eNOS activity. Total eNOS protein was significantly lower (*P* < 0.05) in *eNOS* ^*+/−*^ mice as compared to *eNOS* ^*+/+*^ mice by ~ 60% (Fig. 1C). Despite the marked reduction in eNOS protein, eNOS Ser1176‐phosphorylation levels were significantly higher (*P* < 0.05) in *eNOS* ^*+/−*^ mice under baseline conditions (Fig. 1C).

### Body weight, adiposity, glycemia, and systolic blood pressure in control and high‐fat fed *eNOS* ^*+/+*^ and *eNOS* ^*+/−*^ mice

**Figure 2:**
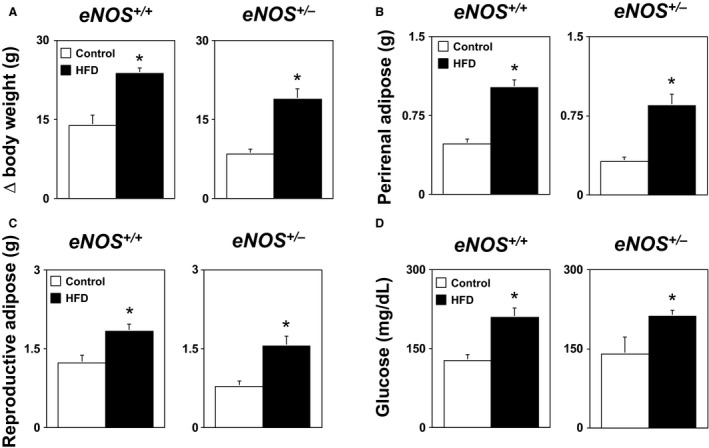
(A) Body weight gain, (B) perirenal adipose mass, (C) reproductive adipose mass, and (D) fasting blood glucose levels in *eNOS* ^*+/+*^ and *eNOS* ^*+/−*^ mice fed either a control or HFD. These findings demonstrate that loss of a single *eNOS* gene is not associated with alterations in baseline metabolic phenotypes or the development of obesity in response to a HFD. Values are Means ± SE;* n* = 6–10/group; *P < 0.05 versus Control.

Body weight was similar (*P* > 0.05) in *eNOS* ^*+/+*^ and *eNOS* ^*+/−*^ mice (22 ± 1 and 23 ± 1 g, respectively) prior to the start of either control or HFD. Body weight gain as well as perirenal and reproductive (epididymal) adipose masses were similar (*P* > 0.05) in *eNOS* ^*+/+*^ and *eNOS* ^*+/−*^ mice fed a control diet (Fig. 2A–C). In contrast, body weight gain and adiposity were significantly greater (*P* < 0.05) in both *eNOS* ^*+/+*^ and *eNOS* ^*+/−*^ mice fed a HFD compared to their respective control diet counterparts (Fig. 2A–C). The degree of obesity and adiposity was not influenced by genotype, as body weight gain and perirenal and reproductive adipose masses were similar (*P* > 0.05) in high‐fat‐fed *eNOS* ^*+/+*^ and *eNOS* ^*+/−*^ mice (Fig. 2B–C).

Blood glucose levels were similar (*P* > 0.05) in *eNOS* ^*+/+*^ and *eNOS* ^*+/−*^ mice fed a control diet (Fig. 2D). In contrast, blood glucose levels were significantly higher in *eNOS* ^*+/+*^ and *eNOS* ^*+/−*^ mice fed a HFD as compared to their control diet counterparts (Fig. 2D). The degree of hyperglycemia produced by a HFD was not influenced by genotype, as the increases in plasma glucose levels produced by a HFD were similar (*P* > 0.05) in *eNOS* ^*+/+*^ and *eNOS* ^*+/−*^ mice (Fig. 2D). Blood pressure was similar in *eNOS* ^*+/+*^ and *eNOS* ^*+/−*^ mice fed a control, and a HFD had no effect (*P* > 0.05) on blood pressure in either *eNOS* ^*+/+*^ or *eNOS* ^*+/−*^ mice (data not shown).

### Responses of carotid arteries from *eNOS* ^*+/+*^ and *eNOS* ^*+/−*^ mice fed a HFD

**Figure 3:**
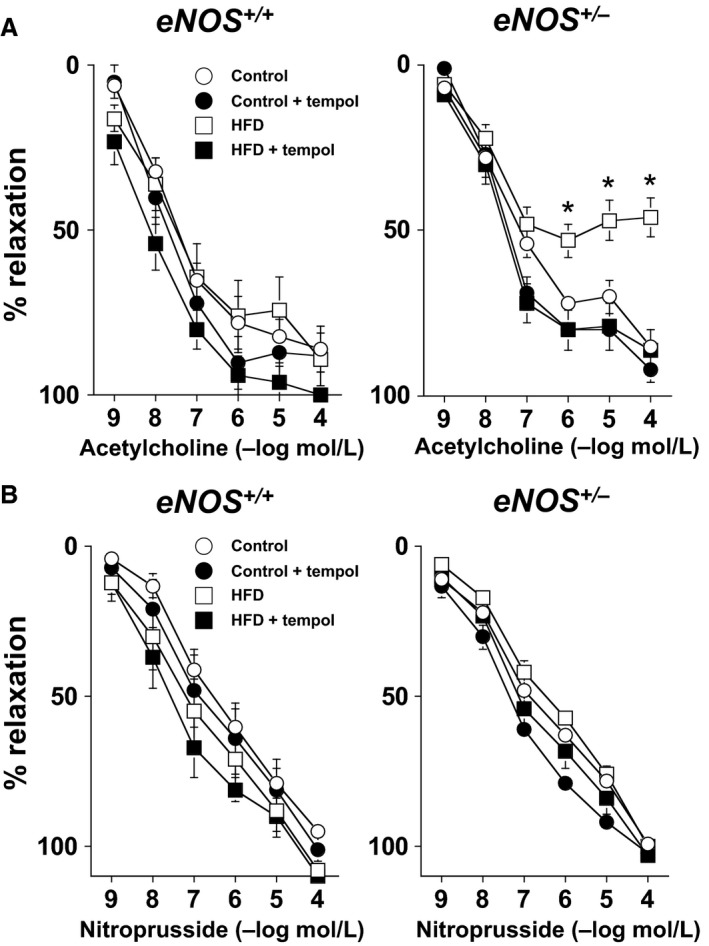
(A) Responses to acetylcholine in *eNOS* ^*+/+*^ and *eNOS* ^*+/−*^ mice fed a control diet or HFD. Endothelial function was selectively impaired in *eNOS* ^*+/−*^ mice fed a HFD. Endothelial function could be improved in carotid arteries from *eNOS* ^*+/−*^ mice fed a HFD treated with Tempol (1 mmol/L) implicating a role for superoxide in the impairment of endothelial function. (B) Reponses to nitroprusside in carotid arteries from *eNOS* ^*+/+*^ and *eNOS* ^*+/−*^ fed either a control or HFD. Responses to nitroprusside were unaffected by genotype, diet, or Tempol, suggesting that the impairment of vascular response to acetylcholine were selective for endothelium. Values are Means ± SE;* n* = 6–10/group *P < 0.05 versus *eNOS* ^*+/−*^ Control and HFD + Tempol.

Acetylcholine produced a similar degree (*P* > 0.05) of relaxation in carotid arteries from *eNOS* ^*+/+*^ and *eNOS* ^*+/−*^ mice fed a control diet. For example, 100 μmol/L acetylcholine produced 86 ± 7% and 86 ± 2% relaxation in *eNOS* ^*+/+*^ and *eNOS* ^*+/−*^ mice, respectively (Fig. 3A). Responses to the endothelium‐independent agonist nitroprusside also produced relaxation that was similar (*P* > 0.05) in carotid arteries from *eNOS* ^*+/+*^ and *eNOS* ^*+/−*^ mice fed a control diet (Fig. 3B).

Responses to acetylcholine were similar (*P* > 0.05) in carotid arteries from high‐fat‐fed *eNOS* ^*+/+*^ mice compared to *eNOS* ^*+/+*^ mice fed a control diet (Fig. 3A). In contrast, responses to acetylcholine were markedly impaired (*P* < 0.05) in carotid arteries from high‐fat‐fed *eNOS* ^*+/−*^ mice as compared to those in either *eNOS* ^*+/+*^ mice fed a control or HFD as well as *eNOS* ^*+/−*^ mice fed a control diet (Fig. 3A). For example, 100 μmol/L acetylcholine produced 86 ± 7% and 46 ± 6% relaxation in *eNOS* ^*+/−*^ mice fed a control or a HFD, respectively.

Tempol had no effect (*P* > 0.05) on vascular responses to acetylcholine or nitroprusside in *eNOS* ^*+/+*^ mice fed a control or HFD (Fig. 3A and B). Tempol also had no effect (*P* > 0.05) on vascular responses in *eNOS* ^*+/−*^ mice fed a control diet (Fig. 3A and B). In contrast, responses to acetylcholine could be significantly improved (*P* < 0.05) in carotid arteries from *eNOS* ^*+/−*^ mice fed a HFD treated with Tempol, implicating a role for superoxide in the impairment of endothelial function in *eNOS* ^*+/−*^ mice fed a HFD (Fig. 3A).

### Vascular eNOS expression and plasma interleukin‐6 levels in *eNOS* ^*+/+*^ and *eNOS* ^*+/−*^ mice fed a HFD

**Figure 4:**
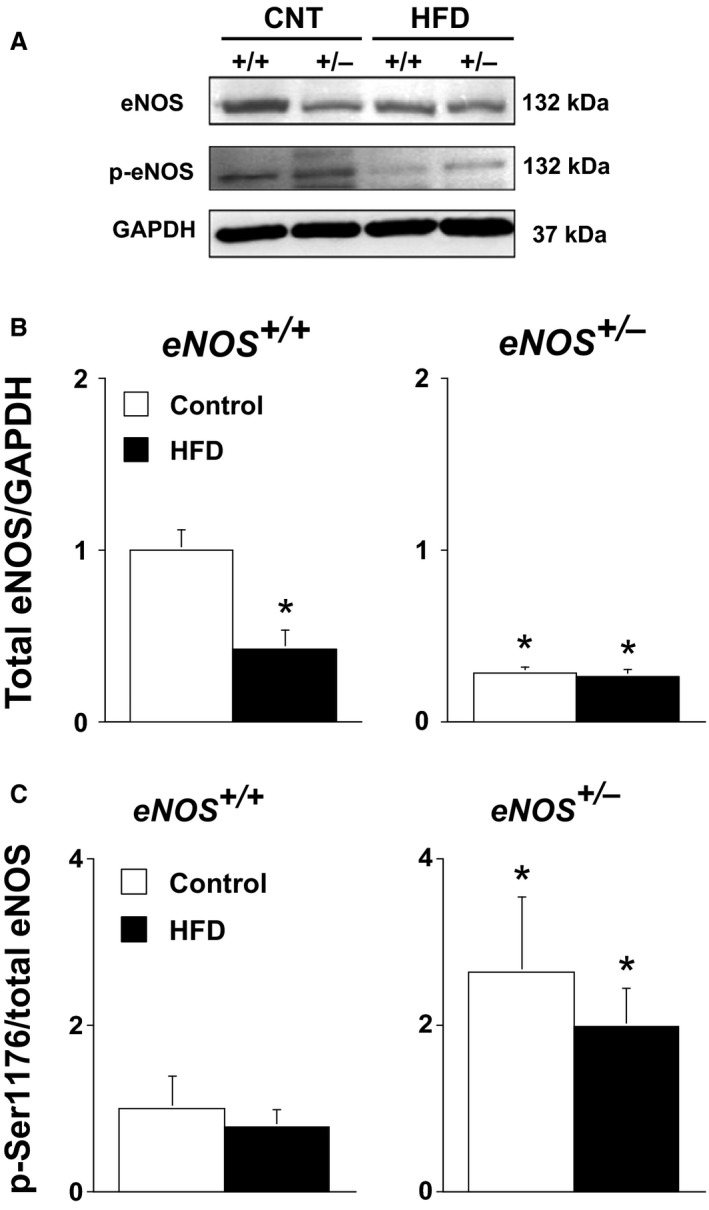
(A) Western blot for total eNOS expression and phosphorylated Ser1176 eNOS levels in aorta form *eNOS* ^*+/+*^ and *eNOS* ^*+/−*^ fed either a control or HFD. (B) Quantification of total eNOS expression, and (C) phosphorylated Ser1176 eNOS levels. Values are Means ± SE;* n* = 5/group; *P < 0.05 versus *eNOS* ^*+/+*^.

Total vascular eNOS levels were significantly lower, i.e., ~60% lower, (*P* < 0.05) in *eNOS* ^*+/−*^ mice compared to that in *eNOS* ^*+/+*^ mice (Fig. 4 and B). A HFD was associated with additional reductions in total eNOS protein levels in both *eNOS* ^*+/+*^ and *eNOS* ^*+/−*^ mice compared to their respective control diet counterparts (Fig. 4A and B).

Phosphorylation levels of eNOS at Ser1176 was significantly higher (*P* < 0.05) in *eNOS* ^*+/−*^ mice than *eNOS* ^*+/+*^ mice fed a control diet (Fig. 4A and C). In contrast, a HFD was associated with lower levels of eNOS Ser1176 phosphorylation in both high‐fat‐fed *eNOS* ^*+/+*^ and *eNOS* ^*+/−*^ mice (Fig. 4A and C).

**Figure 5:**
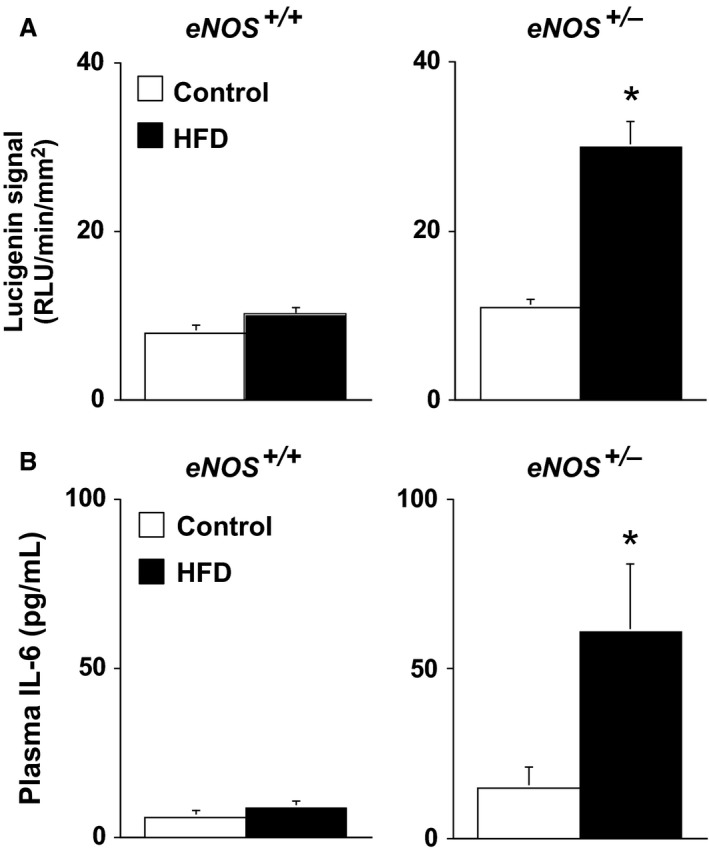
(A) Vascular superoxide levels *eNOS* ^*+/+*^ and *eNOS* ^*+/−*^ fed either a control or HFD. A HFD was associated with a selective increase in vascular superoxide in *eNOS* ^*+/−*^ mice consistent with our functional data. (B) Plasma interleukin‐6 levels in *eNOS* ^*+/+*^ and *eNOS* ^*+/−*^ mice fed either a control or HFD. IL‐6 levels were selectively increased in *eNOS* ^*+/−*^ in response to a HFD and correlated with increases in vascular superoxide. Values are Means ± SE;* n* = 5/group; *P < 0.05 versus *eNOS* ^*+/+*^. ^†^ *P* < 0.05 versus Control.

Superoxide levels were similar (*P* > 0.05) in *eNOS* ^*+/+*^ and *eNOS* ^*+/−*^ mice fed a control diet (Fig. 5A). While a HFD had no effect on superoxide levels in *eNOS* ^*+/+*^ mice fed a HFD, superoxide levels were significantly increased (*P* < 0.05) in *eNOS* ^*+/−*^ mice fed a HFD (Fig. 5A). Plasma IL‐6 levels were relatively low in both eN*OS* ^*+/+*^ and *eNOS* ^*+/−*^ mice fed a control diet (Fig. 5B). In contrast, a HFD was associated with selective increases in plasma IL‐6 levels in *eNOS* ^*+/−*^ (Fig. 5B).

### Exogenous IL‐6 is associated with endothelial dysfunction and increased vascular superoxide levels in *eNOS* ^*+/−*^ mice

**Figure 6:**
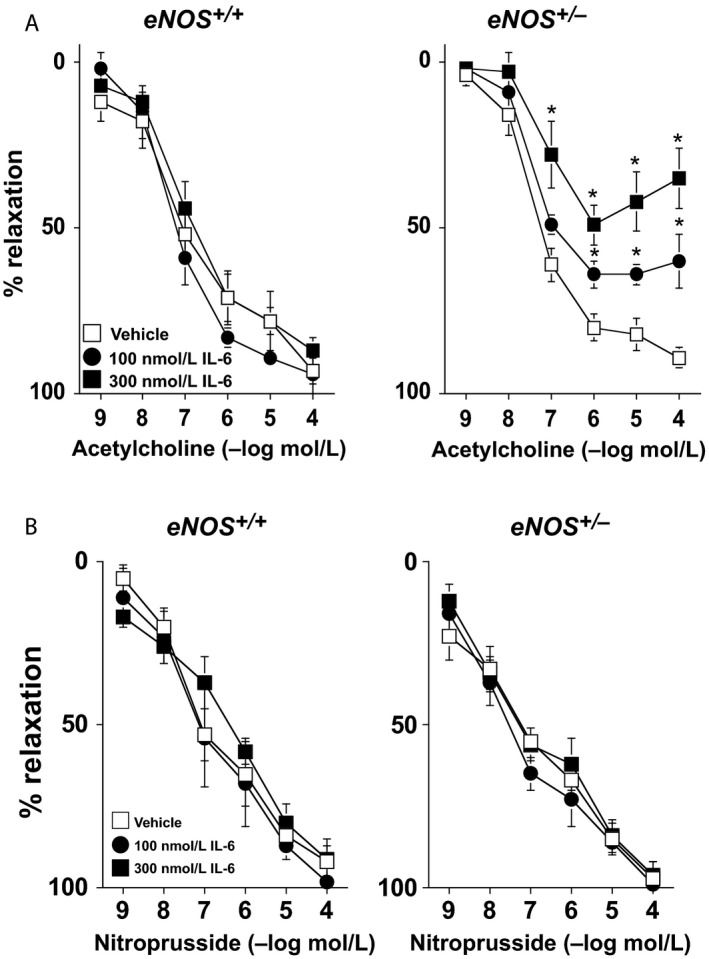
(A) Responses to acetylcholine in carotid arteries from *eNOS* ^*+/+*^ and *eNOS* ^*+/−*^ mice incubated with vehicle (saline) or mouse recombinant IL‐6 (100 or 300 nmol/L). IL‐6 was associated with concentration‐dependent impairment of endothelial function in *eNOS* ^*+/−*^, but not *eNOS* ^*+/+*^, mice. (B) Responses to nitroprusside in carotid arteries from *eNOS* ^*+/+*^ and *eNOS* ^*+/−*^ mice incubated with vehicle (saline) or mouse recombinant IL‐6 (100 or 300 nmol/L). IL‐6 was not associated with alterations in nitroprusside‐induced relaxation, suggesting that the impairment of acetylcholine response by IL‐6 were selective for endothelium. Values are Means ± SE;* n* = 6–8/group; *P < 0.05 versus Vehicle.

In vehicle‐treated carotid arteries, acetylcholine produced a similar degree of relaxation in both *eNOS* ^*+/+*^ and *eNOS* ^*+/−*^ mice (Fig. 6A). Relaxation in response to acetylcholine was not altered in carotid arteries from *eNOS* ^*+/+*^ mice incubated with IL‐6 (Fig. 6A). In contrast, IL‐6 produced concentration‐dependent impairment (*P* < 0.05) of responses to acetylcholine in carotid arteries from *eNOS* ^*+/−*^ mice (Fig. 6A). For example, acetylcholine (100 μmol/L) produced 89 ± 1%, 60 ± 8%, and 35 ± 4% relaxation in arteries from *eNOS* ^*+/−*^ mice treated with vehicle, 100 nmol/L IL‐6, and 300 nmol/L IL‐6, respectively. IL‐6 had no effect (*P* > 0.05) on responses to nitroprusside in either *eNOS* ^*+/+*^ or *eNOS* ^*+/−*^ mice (Fig. 6B).

**Figure 7:**
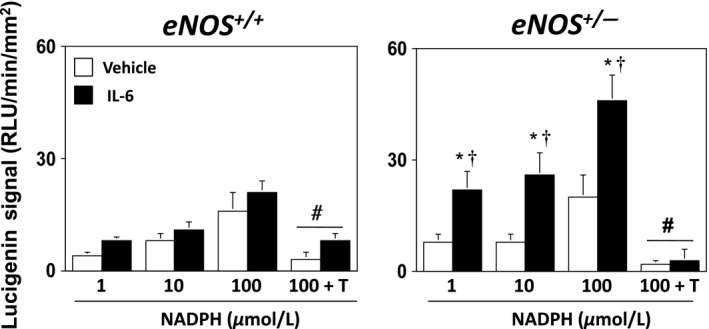
NADPH‐stimulated superoxide levels in aorta from *eNOS* ^*+/+*^ and *eNOS* ^*+/−*^ mice incubated with vehicle or IL‐6 (300 nmol/L). IL‐6 was associated with greater superoxide levels in response to NADPH, suggesting a role for IL‐6‐induced NADPH oxidase‐derived superoxide. Values are Means ± SE;* n* = 6–8/group; T = Tiron; *P < 0.05 versus Vehicle; ^†^ *P* < 0.05 versus *eNOS* ^*+/+*^; ^#^ *P* < 0.05 versus 100 μmol/L NADPH.

Basal superoxide levels were similar in aorta from *eNOS* ^*+/+*^ or *eNOS* ^*+/−*^ mice incubated with vehicle, e.g., superoxide levels were 5 ± 1 and 7 ± 1 RLU/min/mm^2^ in vehicle‐treated vessels from *eNOS* ^*+/+*^ or *eNOS* ^*+/−*^ mice, respectively. NADPH produced concentration‐dependent increases in superoxide levels in vessels from *eNOS* ^*+/+*^ and *eNOS* ^*+/−*^ mice incubated with vehicle (Fig. 7). However, IL‐6 was associated with significantly greater NADPH‐stimulated superoxide levels in *eNOS* ^*+/−*^ mice (Fig. 7). The superoxide signal to 100 μmol/L NADPH in both *eNOS* ^*+/+*^ and *eNOS* ^*+/−*^ mice was abolished with the addition of a superoxide scavenger, thus serving to confirm that the lucigenin signal was due to superoxide (Fig. 7).

## Discussion

There are several new and important findings from this study. First, acetylcholine and nitroprusside were found to produce a similar degree of relaxation in *eNOS* ^*+/−*^ mice to that in *eNOS* ^*+/+*^ mice. Moreover, the vascular responses to acetylcholine in *eNOS* ^*+/−*^ mice are inhibited by L‐NNA and ODQ, suggesting that vascular responses in *eNOS* ^*+/−*^ mice are largely NOS‐ and sGC‐dependent. Second, despite a 60% reduction in eNOS protein expression levels, eNOS activity was greater in *eNOS* ^*+/−*^ mice under baseline conditions, most likely as compensatory response associated with the loss of a single eNOS gene. Third, despite producing similar degrees of obesity and hyperglycemia in *eNOS* ^*+/+*^ and *eNOS* ^*+/−*^ mice, a HFD was only associated with impairment of endothelial responses in *eNOS* ^*+/−*^ mice. Responses to acetylcholine in high‐fat‐fed *eNOS* ^*+/−*^ mice could be improved by Tempol, implicating a role for superoxide in the impairment of endothelial function. Fourth, vascular superoxide and plasma IL‐6 levels were selectively increased in *eNOS* ^*+/−*^ mice fed a HFD as compared to *eNOS* ^*+/+*^ mice fed either a control or a HFD. Fifth, incubation of carotid arteries with exogenous IL‐6 produced concentration‐dependent impairment of endothelial function in vessels from *eNOS* ^*+/−*^, but not *eNOS* ^*+/+*^, mice. IL‐6 also produced marked increases in both basal‐ and NADPH‐stimulated superoxide levels in aorta from *eNOS* ^*+/−*^ mice, providing a mechanistic link between plasma IL‐6 levels and the endothelial dysfunction observed in *eNOS* ^*+/−*^ mice fed a HFD. Taken together, these findings provide pharmacological and genetic evidence that heterozygous eNOS deficiency is not associated with alterations in endothelial function under baseline conditions, due in part, to compensatory increases in eNOS Ser1176‐phosphorylation and maintained dependency on NOS and sGC. In contrast, when coupled with a HFD, heterozygous eNOS deficiency is associated with increases in vascular superoxide and IL‐6, which contribute to the impairment of endothelial function in response to a HFD.

### Baseline vascular responses in *eNOS* ^+/+^ and *eNOS* ^+/*−*^ mice and dependency on NOS and sGC

Endothelium‐dependent responses in a number of blood vessels in the mouse have been shown to be mediated by both NOS and nitric oxide. For example, the responses to acetylcholine in carotid artery are eliminated by pharmacological inhibition of NOS, such as with L‐NNA (Faraci et al. 1998; Didion et al. 2005). Moreover, genetic studies in *eNOS* ^*−/−*^ mice revealed that relaxation to acetylcholine in the aorta and carotid artery are almost exclusively dependent on eNOS and nitric oxide (Huang et al. 1995; Shesely et al. 1996; Faraci et al. 1998; Chataigneau et al. 1999). In this study, we found that acetylcholine produced a similar degree of relaxation in carotid artery from both *eNOS* ^*+/+*^ *and eNOS* ^*+/−*^ mice under baseline conditions, consistent with previous observations (Lamping and Faraci 2001).

Although endothelium‐dependent responses are normal in *eNOS* ^*+/−*^ mice, total vascular eNOS protein levels were ~60% less in *eNOS* ^*+/−*^ compared to that in *eNOS* ^*+/+*^ mice. Perhaps of greater significance, we found that phosphorylation of eNOS Ser1176 was significantly higher in *eNOS* ^*+/−*^ mice compared to *eNOS* ^*+/+*^ mice. This is important, as increased phosphorylation at Ser1176 is a positive regulator of eNOS activity (Fulton et al. 1999). eNOS Ser1176 phosphorylation levels are reflective of increased kinase activity (e.g., Akt and PKA), decreased dephosphorylation by PP2A, and/or alterations in eNOS protein–protein binding with regulatory proteins such as Heat Shock Protein 90 (Mitchel et al. 1999; Searles 2005; Heiss and Dirsch 2014). Because endothelial function is normal in the absence of a single eNOS gene, we would speculate that the increased in eNOS Ser1176 phosphorylation is most likely reflective of reduced PP2A expression/activity as well as a greater abundance of HSP90 as a result of reduced eNOS protein in *eNOS* ^*+/−*^ mice. Future studies will be required to address these possibilities more definitively.

While increased phosphorylation of eNOS Ser1176 may serve an important role in maintaining acetylcholine‐induced relaxation in *eNOS* ^*+/−*^ mice, it is not known whether such responses are mediated by NOS‐ and sGC‐dependent mechanisms like that in *eNOS* ^*+/+*^ mice. Thus, using a pharmacological approach, we examined the role of NOS and sGC in response to acetylcholine and nitroprusside in *eNOS* ^*+/+*^ and *eNOS* ^*+/−*^ mice. In both *eNOS* ^*+/+*^ and *eNOS* ^*+/−*^ mice, relaxation to acetylcholine was largely inhibited in carotid arteries treated with L‐NNA and ODQ, however, at higher concentrations of acetylcholine, the degree of inhibition produced by L‐NNA appeared biphasic and somewhat incomplete, which is not without precedent (Faraci et al. 1998). The nature of this biphasic response may be reflective of the fact that we used a single concentration of L‐NNA, and with higher concentrations, L‐NNA maybe more effective. We should also point out that the majority (75–80%) of the response to acetylcholine was abolished by L‐NNA and ODQ in this study. More importantly, the degree of inhibition produced by L‐NNA was of similar magnitude in *eNOS* ^*+/+*^ and *eNOS* ^*+/−*^ mice, suggesting that the response to acetylcholine in carotid arteries of *eNOS* ^*+/−*^ mice remain, in large part, dependent on both NOS and sGC.

We also found that the sensitivity of carotid arteries from *eNOS* ^*+/+*^ and *eNOS* ^*+/−*^ mice to nitroprusside was increased following NOS inhibition with L‐NNA. These findings are in agreement with previous data suggesting that with the loss of either one of both eNOS genes that responses to NO donors are enhanced (Faraci et al. 1998; Kojda et al. 1999; Brandes et al. 2000; Hussain et al. 2001). It has been suggested that the enhanced response to NO donors reflects compensatory increases in sGC expression and/or activity that occurs in response to reductions in NOS expression and/or activity (Brandes et al. 2000; Hussain et al. 2001). We found that the response to nitroprusside was significantly inhibited by ODQ in arteries from both *eNOS* ^*+/+*^ and *eNOS* ^*+/−*^ mice, however, ODQ was more effective in limiting the response to nitroprusside in *eNOS* ^*+/+*^ mice as that produced in *eNOS* ^*+/−*^ mice. These pharmacological data suggest that the sGC expression and/or activity may be higher in *eNOS* ^*+/−*^ mice, which could, in addition to increased eNOS Ser1176 phosphorylation, serve to support normal responses to acetylcholine and nitroprusside in *eNOS* ^*+/−*^ mice.

### Metabolic phenotypes and obesity development in *eNOS* ^*+/+*^ and *eNOS* ^*+/−*^ mice fed a high fat diet

A HFD is a well‐established model of diet‐induced obesity in mice as it recapitulates many of the characteristics associated with human obesity, including the development of visceral adiposity, insulin resistance, and hyperglycemia (Brownlow et al. 1996; Collins et al. 2004; Lynch et al., 2013; Surwit et al. 1995). In addition to producing a number of metabolic phenotypes, a HFD is also associated with impairment of endothelial function (Molnar et al. 2005; Lynch et al. 2013). For example, a HFD has been shown to produce endothelial dysfunction by at least 12 weeks in cerebral arterioles, whereas 36 weeks of high‐fat feeding is required to produce endothelial dysfunction in the carotid artery, suggesting that a HFD produces endothelial dysfunction in a temporal‐ and vessel‐dependent manner (Lynch et al. 2013). In this study, we elected to study the effects of 30 weeks of high‐fat feeding as we have shown previously that this is a time point that is not associated with impairment of endothelial function in *eNOS* ^*+/+*^ mice fed a HFD (Lynch et al. 2013). Thus, we predicted that a HFD for 30 weeks in *eNOS* ^*+/−*^ mice would be an effective time point to unmask any potential metabolic and/or vascular phenotypes that might be produced in response to a HFD.

While *eNOS* ^*−/−*^ mice display a number of overt phenotypes (Shankar et al. 2000; Duplain et al. 2001; Cook et al. 2004), much less is known regarding metabolic and vascular phenotypes in *eNOS* ^*+/−*^ mice under baseline conditions or in response to a HFD (Cook et al. 2004). This is somewhat surprising as *eNOS* ^*+/−*^ mice have a greater potential to model the impact of human eNOS polymorphisms associated with reductions in eNOS activity and/or nitric oxide on endothelial function (Smithies et al. 2000). In this study, we found that body weight as well as perirenal and reproductive adipose mass were similar in *eNOS* ^*+/−*^ mice fed a control diet compared to *eNOS* ^*+/+*^ mice. Fasting plasma glucose levels were similar and within normal ranges in *eNOS* ^*+/+*^ and *eNOS* ^*+/−*^ mice fed a control diet, suggesting that heterozygous eNOS deficiency per se is not associated with alterations in body weight, adiposity, or plasma glucose levels. A HFD produced marked increases in body weight and visceral adiposity (perirenal and reproductive fat masses) in *eNOS* ^*+/+*^ mice consistent with previous findings (Lynch et al. 2013). Like that in *eNOS* ^*+/+*^ mice, a HFD was associated with increased body weight and visceral adiposity as well as increased fasting glucose levels in the *eNOS* ^*+/−*^ mice in this study. More importantly, the increase in body weight, and perirenal and reproductive fat mass produced in response to a HFD in *eNOS* ^*+/−*^ mice was very similar to that in *eNOS* ^*+/+*^ mice, consistent with the effects of short‐term exposure (<8 weeks) to a HFD (Cook et al. 2004). Collectively, these findings demonstrate that loss of a single eNOS gene is not associated with differences in the development of obesity or hyperglycemia in response to a HFD.

### Endothelial dysfunction in *eNOS* ^+/*−*^ mice fed a HFD is associated with increased plasma IL‐6 and oxidative stress

Obesity, both in humans and a number of animal models, is associated with endothelial dysfunction (Campia et al. 2012; Didion et al. 2005, 2007; Dobrian et al. 2001; Lynch et al. 2013; Molnar et al. 2005). In this study, we found that the vascular responses to acetylcholine were similar in *eNOS* ^*+/+*^ mice fed either a control or HFD for 30 weeks consistent with previous findings at the same time point with the same diet (Lynch et al. 2013). In contrast, responses of carotid arteries to acetylcholine were significantly impaired in *eNOS* ^*+/−*^ mice fed a HFD at a time point that is not associated with impairment of endothelial function in *eNOS* ^*+/+*^ mice. Endothelial function in high‐fat‐fed *eNOS* ^*+/−*^ mice could be significantly improved by Tempol, providing pharmacological evidence for superoxide in the impairment of endothelial responses in HFD fed *eNOS* ^*+/−*^ mice. The effects of a HFD in *eNOS* ^*+/−*^ mice were selective for endothelium, as responses to nitroprusside were not affected by genotype or diet.

Several stimuli are known to influence expression of eNOS (Balligand et al. 2009; Cosentino et al. 1997; Drummond et al. 2000; Saura et al. 2006; Srinivasan et al. 2004; Sun et al. 2012; Yoshizumi et al. 1993). For example, IL‐6, an inflammatory cytokine, has been shown to produce reductions in eNOS expression and activity in endothelial cells in culture in a concentration‐ and time‐dependent manner (Saura et al. 2006). The decrease in eNOS expression produced by IL‐6 involves Stat3‐mediated reductions in eNOS promoter transactivation (Saura et al. 2006). These findings provide at least one mechanism by which IL‐6 can limit NO and impair endothelial function.

In this study, we found that the plasma IL‐6 levels were selectively increased in *eNOS* ^*+/−*^ mice fed a HFD as compared to *eNOS* ^*+/+*^ mice fed either a control diet or a HFD. The increase in plasma IL‐6 levels provides a potential mechanistic link between the selective impairment of endothelial function in *eNOS* ^*+/−*^ mice fed a HFD. However, in of themselves, these findings do not demonstrate causality. In order to directly examine the effect of IL‐6 on vascular function in *eNOS* ^*+/−*^ mice, we treated vessels from *eNOS* ^*+/+*^ *and eNOS* ^*+/−*^ mice with exogenous IL‐6. IL‐6 treatment had no effect on responses to either acetylcholine or nitroprusside in *eNOS* ^*+/+*^ mice, suggesting that the 22 h treatment of IL‐6 at these concentrations has no effect on endothelial function. These findings are consistent with previous findings in which IL‐6 has been shown to have no effect on vascular responses in wild‐type C57BL/6 mice (Schrader et al. 2007). In contrast, IL‐6 produced concentration‐dependent impairment of endothelial responses in carotid arteries from *eNOS* ^*+/−*^ mice, suggesting that in the absence of a single eNOS IL‐6 per se can produce a marked degree of endothelial dysfunction, but not when both eNOS genes are present.

As NADPH oxidase has been implicated as an important source of vascular superoxide (Konior et al. 2014), we examined the effect of IL‐6 treatment on vascular NADPH‐stimulated superoxide levels in *eNOS* ^*+/+*^ *and eNOS* ^*+/−*^ mice. IL‐6 was associated with marked and selective increases in NADPH‐stimulated superoxide levels in *eNOS* ^*+/−*^ mice, suggesting that IL‐6 in the absence of a single eNOS gene results in marked increases in NADPH‐derived superoxide levels with resultant impairment of endothelial function. In contrast, IL‐6 had a very little effect on the vascular NADPH‐stimulated superoxide levels in *eNOS* ^*+/+*^ mice. We have shown previously that angiotensin II‐induced endothelial dysfunction requires expression of both IL‐6 and the Nox2 isoform of NADPH oxidase in the vessel wall (Schrader et al. 2007), the results of this study are supportive of this concept. Exactly, how a HFD promotes increases in IL‐6 in the absence of a single eNOS gene is not clear. NO has been shown to suppress the release of cytokines (DeCaterina et al., 1995), including IL‐6, thus a similar mechanism may play a role in our in vivo studies. Most likely, any suppressive role that NO exerts on IL‐6 release in this study appears to occur independently of obesity or hyperglycemia. Our ex vivo reconstitution experiments with IL‐6 suggests that the effects of IL‐6 occur within the vessel wall.

## Conclusions

In summary, these findings demonstrate that despite an ~60% reduction in eNOS protein expression endothelial function in *eNOS* ^*+/−*^ mice is normal and NOS‐ and sGC‐dependent due, in part, to a compensatory increase in eNOS Ser1176‐phosphorylation. Our findings also demonstrate that while the development of obesity and related metabolic phenotypes in response to a HFD are not affected by eNOS genotype, endothelial function is selectively impaired in *eNOS* ^*+/−*^ mice fed a HFD, and is associated with increases in plasma IL‐6 and vascular superoxide. Perhaps most importantly, our reconstitution experiments serve to mechanistically link the increase in plasma IL‐6 levels produced by a HFD with the impairment of endothelial function observed in *eNOS* ^*+/−*^ mice. Our findings are particularly relevant clinically as they are an example of eNOS haploinsufficiency and as endothelial dysfunction is a major risk factor for carotid artery disease and ischemic stroke (Den Ruijter et al. 2012; Kappus et al. 2014).

## Conflict of Interest

None declared.
